# Pre-clinical development of a wireless neural interface system for osseointegrated prosthetic control in sheep

**DOI:** 10.3389/fnins.2025.1681136

**Published:** 2025-11-06

**Authors:** Lucas Sears, Ashlesha Deshmukh, Rishith Mereddy, Vinson Go, Brett Nemke, Yan Lu, Weifeng Zeng, Aaron J. Suminski, Mark Markel, Kent N. Bachus, James Morizio, Sam O. Poore, Aaron M. Dingle

**Affiliations:** 1 Division of Plastic Surgery, University of Wisconsin, Madison, WI, United States; 2 Wisconsin Institute for Translational Neuroengineering, University of Wisconsin, Madison, WI, United States; 3 Department of Electrical and Computer Engineering, Pratt School of Engineering, Duke University, Durham, NC, United States; 4 School of Veterinary Medicine, University of Wisconsin, Madison, WI, United States; 5 Department of Neurological Surgery, University of Wisconsin, Madison, WI, United States; 6 Department of Orthopedics, School of Medicine, University of Utah, Salt Lake City, UT, United States; 7 George E. Wahlen Department of Veterans Affairs Medical Center, Salt Lake City, UT, United States; 8 William S. Middleton Memorial Veterans Hospital, Madison, WI, United States

**Keywords:** peripheral nerve interface, large animal model, osseointegration, surgical methodology, telemetry

## Abstract

The Osseointegrated Neural Interface (ONI) is an innovative peripheral nerve interface design that houses a transected nerve and coupled electrical components within the medullary canal of long bones for eventual prosthetic control. Before the ONI can enter clinical testing, it must demonstrate longitudinal durability in an animal model analogous to the human anatomy. Adult sheep, possessing comparable weight and bone structure to adult humans, serve as the standard model for osseointegration research, solidifying them as the ideal animal for the development of an ONI. In this paper, we introduce an Ovine ONI model with a wireless, dual capsule implantable peripheral nerve interface capable of remote stimulation and recording of our subject’s nervous system 8 weeks post-implantation. This study investigates the interface design, surgical methodology, radiological evidence, and electrophysiological data that substantiate the osseointegrated approach to interfacing with the peripheral nervous system. We also explore the functional specifications, 3D printing, and coating processing steps for the capsule. Furthermore, our exploration includes the post-processing data analysis methodology used to validate our interface. This methodological study not only contributes crucial insights but also establishes the essential foundation for future goals of the ONI project. Emphasizing real-world applicability through closed-loop interfacing and enhanced efficacy of recording devices.

## Introduction

Amputation is a globally prevalent procedure which often places a substantial physical and psychosocial burden on patients (McDonald et al., 2021). While contemporary prosthetic rehabilitation addresses the physical absence of limbs, the lack of sensory feedback and response to user input fails to satisfy critical domains of prosthetic embodiment such as ownership and agency. Ownership entails integration of the prosthetic device into the user’s own body schema, while agency requires that device outputs represent an extension of the user’s intentions; both domains are contingent on an artificial limb that provides adequate sensory feedback while responding congruently with patient input (Zbinden et al., 2022). Non-electronic prosthetics fall short of these demands due to their minimal sensory feedback and non-intuitive methods of control. To overcome these limitations, the forthcoming field of neural prosthetics aims to interface directly with the patient’s nervous system, enabling fluid sensory feedback and motor control of a prosthetic device.

Integration of a neural prosthetic with the patient’s nervous system is widely achieved through a combination of external or implantable electrodes, integrated circuit boards, power supply, and software all concerted to translate neural or environmental input into prosthetic movement or sensory stimulation. Interface can be achieved at the level of the peripheral or central nervous system, with brain computer interfaces boasting the potential for greater selectivity than peripheral nerve interfaces—but often at the cost of more stringent performance demands and a loss of chronic stability (Ghafoor et al., 2017; Yildiz et al., 2020). Regardless of the interface location, contemporary neuroprosthetic limbs are hamstrung by limitations in device design and manufacturing; challenges involve balancing the selectivity and stability of implanted components, managing the formation of chronically painful neuromas, and satisfying the telemetric demands of high-density neural recording data.

From the neural recording and stimulating perspective, surface electromyogram (EMG) provides a non-invasive alternative to percutaneous and transcutaneous recording electrodes, but the constrained selectivity of this approach renders it impractical for use in a sophisticated prosthetic. Furthermore, the limited proximity of surface electrodes to target nerves introduces substantial noise into recording data and precludes their use in afferent stimulation of the nervous system. Conversely, implanted electronics boast superior selectivity through their proximity to target nerves but carry with them an ever-present risk of infection, contraindicating their use in a longitudinal context (Tyler, 2018). Balancing the selectivity and invasivity of these systems connects to a primary concern of neuromodulatory devices: chronic biocompatibility. Factors such as foreign body reactions, micromotion within soft tissue interfaces, and even regeneration of tissue damaged in surgery can impede electrical interface with target nerves, completely undermining the function of the neural prosthetic device. Therefore, material selection, surgical methodology, and interface location must be designed around these constraints (Kumosa, 2023).

In a post-amputation context, the condition of damaged nerves becomes a factor of paramount concern; if ignored, a transected nerve will regenerate in an aberrant fashion, resulting in a chronically painful neuroma (Sehirlioglu et al., 2009). Methods of reemploying the residual nerve such as targeted muscular reinnervation (TMR) and the regenerative peripheral nerve interface (RPNI) reduce symptomatic neuroma formation via provision of a viable end target for healthy regeneration in the form of denervated muscle; this reinnervated tissue also serves as a bioamplifier for activity within the target nerve, increasing the specificity of neural recording (Mauch et al., 2023). Despite their numerous advantages, TMR and RPNI do not circumvent the challenges associated with implanting interface components within soft tissue. Furthermore, once interface with the nervous system is achieved, the transfer of high-fidelity recording data to computational components or external data acquisition (DAQ) software demands a device with efficient and robust telemetric capabilities. If a wireless recording system is not able to transmit large amounts of data with minimal power draw, establishment of a real-time bidirectional interface is rendered impossible (Kuan et al., 2015).

**FIGURE 1:**
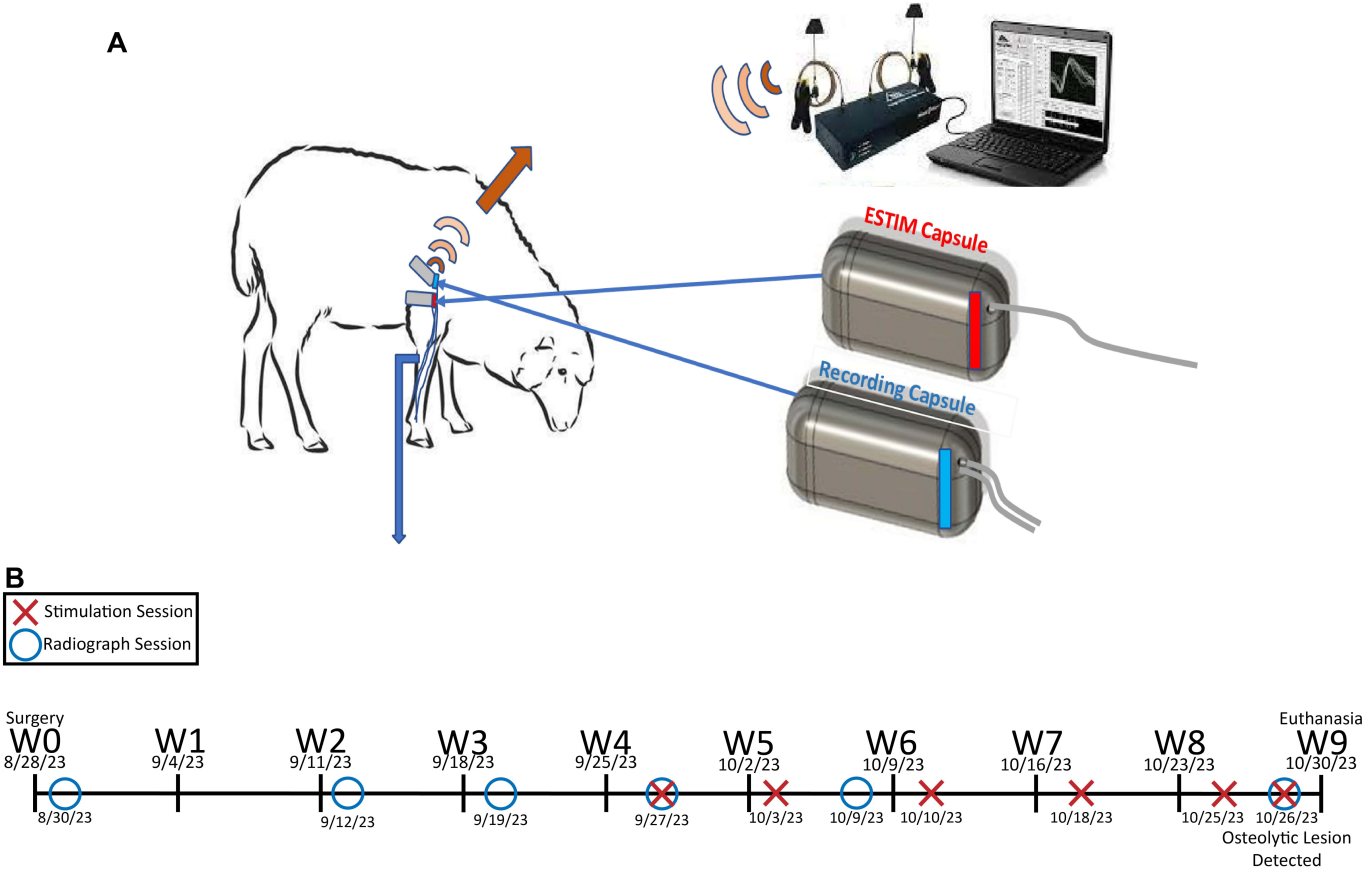
**(A)** Depiction of the general experimental paradigm employed in this study. **(B)** Experimental timeline for Sheep A from week 0 (W0) to week 9 (W9).

Prompted by these obstacles, the Osseointegrated Neural Interface (ONI) seeks to eventually transcend modern prosthetic design through innovative surgical techniques and a neural interface capable of real-time bidirectional control. Featuring two primary components: an endoprosthetic abutment fixed into a reamed medullary canal and an implanted peripheral nerve interface, the ONI subverts traditional peripheral nerve interface (PNI) design by housing the terminal end of a transected nerve and associated electronics within a long bone (Figure 1). The stem cell rich environment of the medullary canal minimizes the formation of painful neuromas, while the surrounding diaphysis mechanically insulates the nerve-electrode unit, aiming to promote greater selectivity in recording and stimulation (Boldrey, 1943). Prior to human implementation, the ONI must demonstrate longitudinal durability in an animal model. Despite the ONI’s previous validation in a rabbit model, human implementation would require success in an animal that better reflects human anatomy, biomechanics and scale (Gunderson et al., 2023; Jeyapalina et al., 2017; Karczewski et al., 2022). Therefore, sheep were selected for this study due to their size and morphological similarity to human beings, particularly in the context of long-bone anatomy (Dingle et al., 2020). More specifically, we implemented an already established ovine trans metacarpal amputation model with osseointegration in which to test the neural interfacing componentry (Jeyapalina et al., 2017).

Here, we demonstrate the feasibility of a wireless Ovine ONI model, introducing radiological and electrophysiological evidence acquired from a mature non-lactating female sheep, as well as radiological evidence from another adult female sheep who underwent an identical surgical procedure with osseointegration and implantation of an inactive peripheral nerve interface with electrical components removed. For clarity, the animal implanted with the active neural interface is referred to as Sheep A, while the animal implanted with the inactive interface is referred to as Sheep B throughout this manuscript. Furthermore, we will explore the osseointegrated abutment, surgical methodology, and dual capsule implantable neural interface characterizing our model, deconstructing the discrete components enabling wireless transfer of neural recording data from our subject to our external DAQ.

## Materials

### Endo- and exo-prosthetic components

**FIGURE 2:**
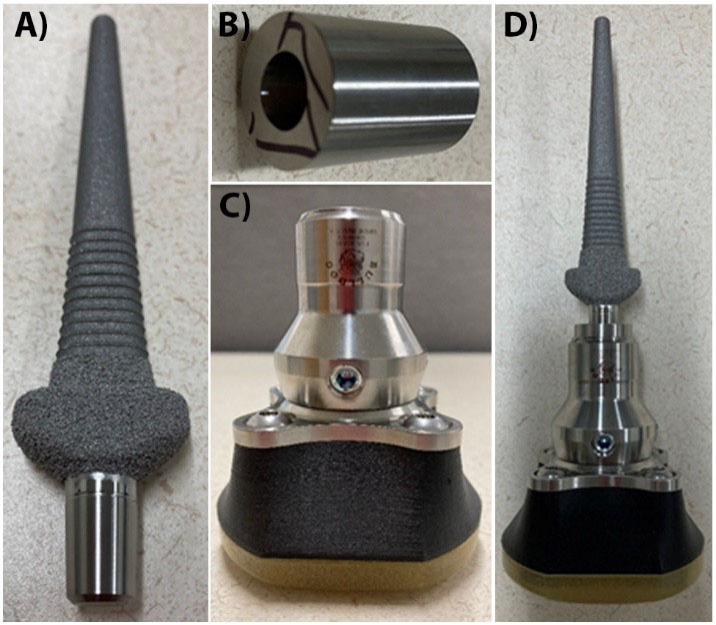
Endo- and exo-prosthetic componentry. The endoprosthetic implant **(A)** is inserted into the medullary canal of the metacarpal bone. The Morse Taper cylinder adapter **(B)** attaches to the percutaneous, smooth portion of the endoprosthesis. The exoprosthetic hoof **(C)** attaches to the cylinder portion of the Morse Taper adapter. The entire endo- and exo- prosthetic construct is shown in **(D)**. Not to scale.

The mechanical components of our prosthetic device consisted of a percutaneous endoprosthesis implanted within the medullary canal of our sheep’s metacarpal bone and a detachable exoprosthetic hoof as previously described (Figure 2; Jeyapalina et al., 2017). The endoprosthesis was fabricated (Avalign Thortex, Portland, OR, USA) from medical grade Ti6A14V titanium alloy. The intramedullary surface of the implant was textured by grit blasting to facilitate osseointegration and a commercially pure titanium (p^2^ type) porous coating (500–750 μm thick with reported porosity of 52 ± 12%) was applied (DJO Global, Austin, TX, USA) to the subdermal barrier (Jeyapalina et al., 2017). The Ti6A14V endoprosthetic was packed in double peel-packs and autoclaved using standard surgical implant autoclave protocols. The Morse Taper cylinder adapter was custom fabricated (Avalign Thortex) and is secured via the Morse Taper to the endoprosthesis, which allows donning and doffing of the exoprosthetic via a cylinder to pyramid adapter (bulldog Tools Inc., Lewisburg, OH, USA). The detachable exoprosthetic hooves were custom fabricated by the Orthopedic Research Laboratory, Department of Orthopedics, University of Utah School of Medicine, Salt Lake City, UT. Proceeding proximally to distally, the manufactured Morse Taper to cylinder adapter fits into a purchased cylinder to pyramid adapter (Bulldog Tools Inc.). This adapter fits onto a purchased pyramid plate (Bulldog Tools Inc.) which proceeds to a custom Onyx (Markforged, Watertown, MA, USA) 3D printed spacer, ending in a custom polyurethane footpad.

### Implantable electronics

**FIGURE 3:**
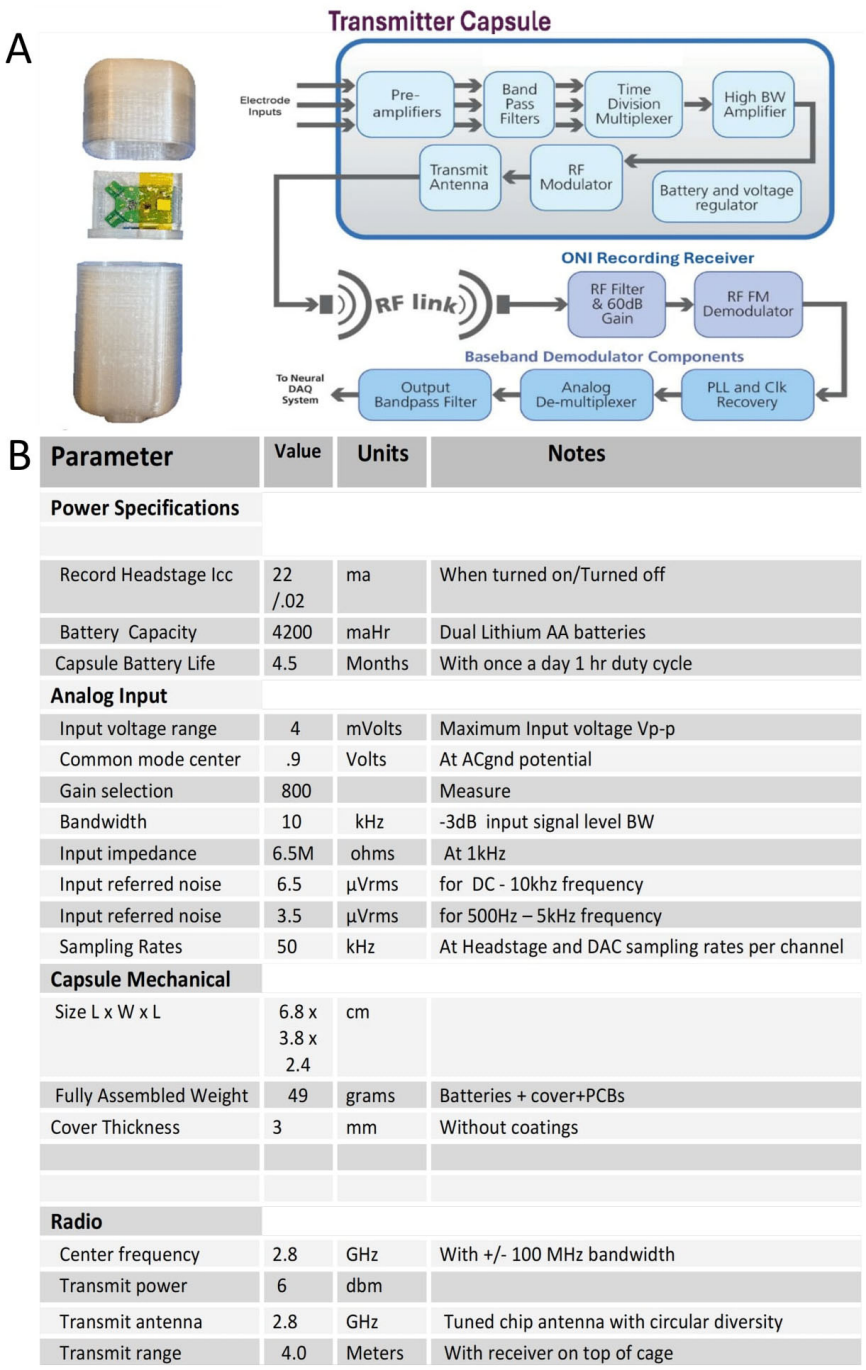
**(A)** Open capsule and system block diagram of the recording transmitter capsule. **(B)** Specification table for the recording capsule.

**FIGURE 4:**
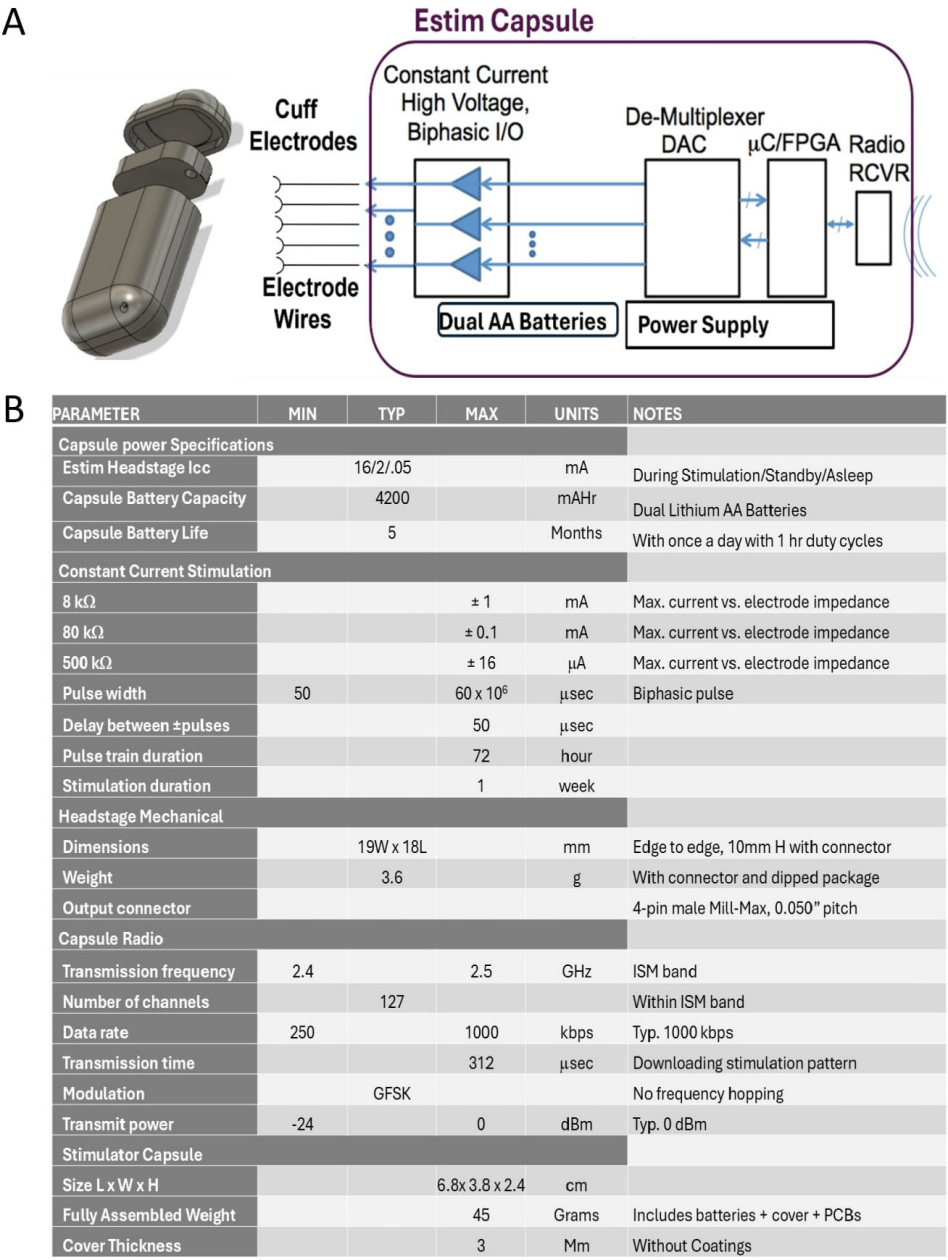
**(A)** 3D drawing and system block diagram of the Estim capsule. **(B)** Specification table for the Estim Capsule.

Electrophysiology was performed through two, separate implantable capsules which were designated to exclusively record or stimulate (Figures 3, 4). This functional separation warranted a simplified approach to power supply and battery protection when compared to a single capsule approach, further protecting the sensitive electronics from fluid infiltrate (Deshmukh et al., 2020). Capsule exteriors were printed using high performance polyethylene terephthalate (PET) filament; a material commonly utilized in other implantable technologies such as valvular grafts, stents, and sutures (Scott, 2023). While known for its low moisture permeability, biocompatibility, and chemically inert nature, PET is sensitive to temperatures above 214° C, precluding the use of autoclave sterilization. Ethylene oxide (EtO) sterilization was employed using standard surgical implant autoclave protocols. Once printed and assembled, the capsules are subject to one coat of medical grade sterile epoxy and two coatings of biocompatible silicone. This process parallels other medical grade human implantable devices and provides a robust defense against the permeation of body fluids. To prevent mechanical damage and moisture breach, extruding electrode and ground wires were housed within silicone tubing throughout their transit to distal cuff electrodes and recording sites. A Microprobes Platinum/Iridium nerve cuff bipolar electrode was employed at the distal end of both implanted capsules, enabling direct interface with the epineurium of our target nerve. Power was supplied by two AA size batteries whose output was mediated via a magnetic switch mechanism; once implanted, the magnetic switch would enable non-invasive power toggling of the implants via a high-powered magnet external to the animal.

### Recording capsule

The recording capsule electronics consisted of a 5-Channel application specific integrated circuit (ASIC) chip mounted on preamplifier-multiplexer printed circuit board (PCB) with five recording electrode wiring inputs, a radio frequency (RF) radio PCB and dual battery power supply (Figure 2).

The five electrode inputs consisted of a reference channel, reference ground, an EMG channel, and a two-channel contribution from the cuff electrode. The analog ASIC will pre-process each analog input channel with a low noise pre-amplifier with gain of 150 and bandpass filtering of 0.3 Hz to 10 k Hz. Each of the 5-Channels are time division multiplexing with a 50 k Hz sampling rate per channel and sent to a custom Voltage Controlled Oscillator (VCO) to produce a broadband frequency modulation (FM) radio signal. The broad band 2.8 gigahertz (GHz) FM radio signal is then sent to a circularized dual chip antennae to foster radio signal integrity independent of record capsule orientation.

### Estim capsule

The Estim capsule housed a wireless headstage PCB, dual AA size battery power supply, and three electrode outputs.

The PCB packaged constant current output drivers, a microcontroller for programmable Estim pulse pattern storage that includes pulse amplitude and pulse timing and a 2.4 Ghz digital radio based on a simplified wireless fidelity (Wi-Fi) telemetry system. Two output channels supplied biphasic constant current pulse that were dedicated to the cuff electrode contacts, conserving the final output for grounding. Stimulation parameters current amplitude and timing were pre-programmed and triggered on a laptop using in-house software and delivered to the implant via a USB transceiver dongle.

### Receiver box

The receiver system was composed of a 2.8 GHz W5 receiver and two associated highly-tuned passive RF antennae. The tuned receiver unit will demodulate the FM signal and demultiplex each of the 5 channels with a total voltage gain of 800 with 0.5 Hz to 10 k Hz bandpass filter bandwidth. The DAQ PCB in the receiver will digitize each of the 5 channels with 16-bit resolution. This system was then coupled via USB with a laptop running in-house software capable of deconstructing and visualizing the multiplexed radio signal transmit by the recording implant. Received data is then saved for further post-processing in NeuroExplorer and MATLAB.

### Cuff electrodes

Cuff electrodes utilized on both the Estim Capsule and Recording Capsule consisted of 1.5 mm inner diameter with 500 micrometer (μm) width platinum-iridium ribbon. Contacts were platinum/iridium, 1 mm wide X 4.7 mm long with a distance of 3.5 mm between contacts with 10.5 mm length between the first/last contact to the edge of the cuff (MicroProbes for Life Science Inc. Gaithersburg, MD, USA, item XWNC-1.5-2-PI-3.5-10.5-SUT-400-00).

### Water ingress testing

The capsules were evaluated using a low-pressure, vacuum-based moisture leakage testing platform designed to mechanically stress the capsule and assess the integrity of the 3D-printed and coated capsule technology. A leakage test was initiated on February 15th, during which the capsule was submerged in saline. The capsule demonstrated excellent performance throughout the testing period, showing no signs of moisture ingress. After 104 days of continuous submersion, no evidence of leakage or bubble formation was observed, even when the capsule was subjected to an internal air pressure differential of −35 psi.

## Methods

### Surgical methodology

#### Ethics statement

All animal procedures were approved by the University of Wisconsin Institutional Animal Care and Use Committee (ICACU #V006200) and the William S. Middleton Memorial Veterans Affairs Animal Component of Research Protocol (ACORP). Defined pre-specified endpoints were prolonged loss of connection with wireless implants OR Pain or distress that cannot be reasonably treated by veterinary intervention (USDA, category D), respectively.

#### Anesthesia

Prior to surgery the sheep were fasted for 24 h and water was withheld for 12 h. Sheep were administered a dose of Xylazine (0.1 to 0.22 mg/kg IM) in their pen to sedate them so they could be moved to the pre-op prep area. Sheep were then induced with ketamine-midazolam (2 to 10 mg/kg to 0.1 to 0.3 mg/kg IM) and immediately intubated. Once intubated the sheep were maintained on isoflurane (0% to 4%) in 100% O2. Intraoperative analgesia was provided by a Morphine, Lidocaine, Ketamine (MLK) (In 1 L bag of saline or LRS 48 mg morphine, 120 mg ketamine, and 600 mg lidocaine) in a continuous rate infusion (CRI) (3–5 mg/kg/h). A loading dose of morphine (0.1 mg/kg IV) and lidocaine (0.5 mg/kg IV) was given immediately prior to surgery. Gentamicin (6.6 mg/kg IV) and K-penicillin (22,000 IU/kg IV) were also given at this time as pre-op antibiotics. The wool over the surgical sites were clipped, surgical sites cleaned, and the animal was transported to the surgical suite. Surgical sites were then aseptically cleaned and draped for surgery using aseptic techniques. The surgical operation begins proximally with a large incision in the sheep’s shoulder for capsule placement and progresses distally with metacarpal amputation and osseointegration followed by transposition of the target nerve and a coupled cuff electrode into the medullary canal. When planning the amputation, special consideration must be made to the proposed location of the neural interface within the reamed out metacarpal, as there needs to be sufficient space to avoid interference between the endoprosthesis and sensitive nerve-electrode unit.

#### Capsule placement

Surgery begins with a 12 cm incision superficial to the sheep’s scapula; sharp and blunt dissection clears out a 12 × 12 cm subcutaneous pocket for capsule housing. Hemostasis must be achieved through electrocautery or meticulous ligation. The capsules are then placed with their wiring oriented distally along the sheep’s forelimb; it is critical to ensure sufficient wire length to connect the electrodes with their end targets. Once settled upon a location, the capsules are secured with 3–0 vicryl sutures to prevent any unwanted micromotion. Two sequential 2 cm incisions are made from the shoulder to the forelimb 15 cm apart from one another; these will enable the wiring to pass subcutaneously from the shoulder to the forelimb. A metal tube was implemented to safely escort the wiring through these new incisions and into the forelimb.

#### Amputation

Amputation begins with a vertical incision on the anterior side of the metacarpal joint, preserving the skin flap posteriorly. Any tendons connecting the foot are to be transected, and subcutaneous tissue is cleared until the metacarpal can be clearly visualized. With the metacarpal exposed, a saw is used to perform a transverse-osteotomy at the pre-planned level. A K-wire, 7-mm cannulated drill bit, and bone rasp were inserted into the medullary canal, enlarging the diameter until an appropriately sized endoprosthetic can be tightly secured using a slap-hammer.

Proximal to the amputation, a 12–15 cm lazy-S shaped incision is placed beginning on the dorsal metacarpal and ending on the medial side of the animal’s forelimb. The underlying loose areolar tissue is dissected to reveal the primary interdigital sensory nerves of the forelimb, representing branches of the superficial radial nerve. The Central Branch of the Superficial Radial Nerve is the thickest, least variable branch, and is easily recognizable due to its regular proximity to the cephalic vein, cementing it as an ideal target nerve for the interface (Gunderson et al., 2023). The target nerve is then dissected retrograde 10 cm proximal from the wrist for placement of the proximal electrode and anterograde 3 cm distal to the wrist for transposition. Soft tissue overlying the bone is cleared with a hemostat and elevator. A corticotomy is then made 10 cm distal to the carpal joint on the medial side of the bone using a 3/16th inch drill bit and a hand-held electric drill.

#### Electrode interface

**FIGURE 5:**
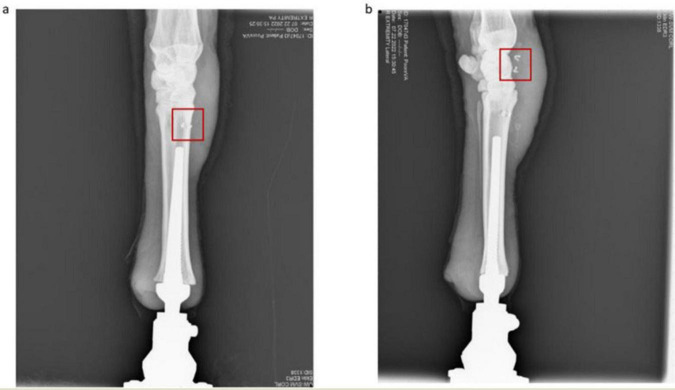
**(a)** Anterior view of osseointegrated ovine thoracic limb for Sheep A. The osseointegrated implant is nested within the reamed medullary canal and the intramedullary electrodes are outlined in red. **(b)** Lateral view of osseointegrated ovine thoracic limb. The recording electrodes proximal to the corticotomy are outlined in red.

Numerous electrodes extrude from the capsules’ wiring. The stimulating capsule has one cuff with two electrode contacts and one reference ground wire electrode, while the recording capsule has one cuff with two electrode contacts and three wire electrodes—reference, reference ground, and EMG. The stimulating cuff electrode is attached to the distal end of the transected target nerve, and the recording cuff is attached 10 cm proximally at a point of minimal tension. The target nerve, coupled with the stimulating cuff, is then transposed into the medullary canal via the corticotomy followed by the reference wire electrode from the recording capsule (Figure 5). Furthermore, the reference ground wire electrode from the stimulation capsule is placed in nearby subcutaneous tissue external to the corticotomy. The recording capsule’s EMG wire electrode is secured in a forelimb muscle proximal to the neural interface, and the reference ground wire should be placed in subcutaneous tissue unoccupied by other electrodes. Finally, prolene suture is employed to secure the silicone tubing to the periosteum, and the capsules are powered on to ensure functionality. 3–0 vicryl sutures are used to close all wounds. The limb was cleaned and bandaged. Electrode interfacing was only performed for Sheep A; Sheep B received an inactive implant with only one wire transposed into the medullary canal via corticotomy which was not secured to a peripheral nerve. This unsecured wire eventually regressed out of the corticotomy.

### Post-operative care

Post-operation, the recovering animal was singly housed to reduce running and the possibility of blunt trauma from other animals. Companion animals were kept in an immediately adjacent pen allowing direct interaction with other sheep. Sheep were housed indoors with controlled temperature and light cycles in a restricted area to minimize opportunistic infections. Post-procedure, animals were immediately assessed (within 1 h) for painful behaviors then assessed at least 3–4 times per day (at least 6–8 h between assessments) for the first 48 h then at least 2–3 per day (at least 8 h between assessments) for 3 further days, then twice a day to 2 weeks, then once a day to 3 weeks post-op using our modified pain scoring system with clear intervention levels noted (Silva et al., 2022). K-Penicillin (22,000 IU/kg IV) was given quarterly 6 h for 7 days post-op. Gentamicin, 6.6 mg/Kg IV was given quarterly 24 h for 7 days. Meloxicam (2 mg/kg on first day, then 1 mg/kg for following days orally) was given once at time of recovery, then at 24 h. intervals for 5 days post-op, then every 48 h to 10 days post-op. Gabapentin was given (0–20 mg/kg Orally) twice daily (BID) as needed during the time that Meloxicam was given. Pantoprazole (1 mg/kg IV, or 2 mg/kg SQ) was given daily with Meloxicam. Fentanyl patch: 25–75 mcg/h. Patch(s) are placed at the end of surgery and replaced every 48 h for up to 8 days post-op. Ketamine (0.5–1 mg/kg IM) was given BID to QID as needed. To ensure proper bone repair, radiological imaging was performed approximately every 2 weeks for the first 12 weeks and then intermittently as needed, visualizing both the medial/lateral and anterior/posterior planes using a portable digital radiography system including an Eklin EDR3 with Canon CXDI-31 plate and a Min-X HF100/30+ generator (VetRocket, Santa Clara, CA, USA). Bandaging was changed every 3 days to assess wound healing and limb perfusion over a 4-week period at which point bandaging ceased. Feeding was left unchanged and adhered to USDA guidelines.

### Electrophysiology

Electrophysiology was only performed in Sheep A, *n* = 1. A period of 4 weeks post operation was observed before the electrophysiology experiments were performed. Electrophysiology was intended to be performed until failure of the telemetric system, which served as the prespecified endpoint for experimentation. Standard procedure saw the animal in its pen with an accompanying researcher who provided close observation and reorientation of the subject when necessary; an additional researcher remained outside of the pen with laptop, receiver box, and antennae. Implants were powered on using a strong magnet external to the animal, and their activity was confirmed via cross communication with our in-house software. Once powered-on, pre-programmed stimulation parameters would be delivered to the stimulation capsule and executed at the intramedullary stimulating cuff electrode. Changes in voltage would be picked up by the extramedullary and intramedullary recording cuff electrodes as well as other supporting recording electrodes to be communicated to the receiver box via radio output of the recording implant. Recorded data was then visualized on our in-house DAQ and saved for further post-processing analysis. Stimulation parameters included amplitude in microamperes (μA), frequency, and duration in seconds; frequency and duration remained at 25 hz and 30 s, respectively, while amplitude was titrated up from 200 to 2,000 μA. Data was recorded in 60 s windows, with no stimulation delivered in the first 20 and last 10 s to establish a baseline of activity.

### Data analysis

Recording data was first imported into NeuroExplorer for qualitative observation of activity within the 60 s session; recording windows with excessive noise were deemed unfit for further analysis and excluded. All other recording sessions were then imported into MATLAB for post-processing, which involved multiple signal processing techniques to enhance the signal-to-noise ratio. A bandpass filter was applied, using 2nd-order infinite impulse response Butterworth filter and a passband of 30 to 10,000 Hz. For the removal of powerline interference, we employed notch filters at 60 Hz and its harmonics (120, 180, 240, and 300 Hz) using 2nd-order Butterworth bandstop filters. Next, we performed reference channel subtraction, where the reference signal was subtracted from the neural channels to remove common-mode noise. Finally, we applied Gaussian smoothing of 20 data points window size and a standard deviation of approximately 3.33 employing a moving average filter with Gaussian weights to remove high-frequency noise while preserving stimulation artifact.

## Results

Postoperation, sheep were closely observed to ensure proper behavioral and physical recovery following implantation and osseointegration. Accompanying our qualitative observations, radiographs were taken of the recovering forelimb to visualize the repair process and validate the successful placement of our cuff electrodes. Four weeks post-implantation, Sheep A was fully weight-bearing and freely ambulating on the prosthetic limb; her behavior was recorded as bright, alert, and responsive. Successful osseointegration was qualitatively inferred from pain free ambulation with weight-bearing on the prosthetic and the absence of appreciable changes in implant position from week to week.

**FIGURE 6:**
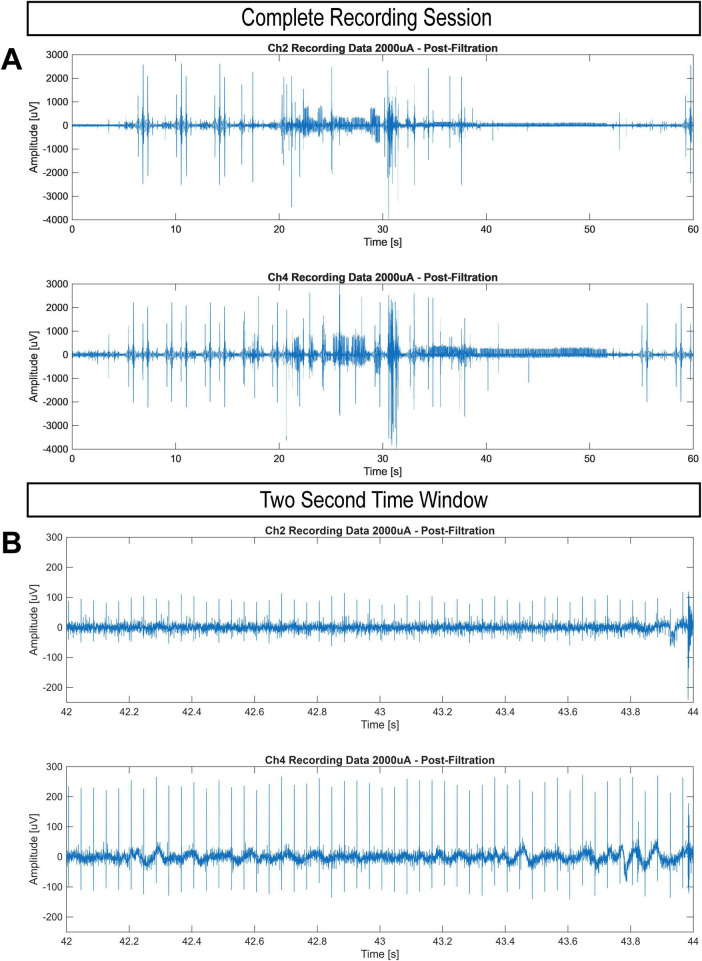
Dual-Channel recordings of a stimulation session delivering 2,000 μA at 25 Hz for 30 s (20–50 s) approximately four weeks post-surgery. Channel 2 represents the extra-medullary recording electrode while Channel 4 represents the intra-medullary recording electrode. All data underwent filtration pipeline detailed in the methods section. **(A)** Complete 60 s recording window. **(B)** Two second time window which illustrates the difference in signal strength between the two electrodes.

A positive recovery trajectory motivated the beginning of our electrophysiological experiments. Analysis of our first stimulation and recording session elucidated the presence of artifact produced by our stimulating electrode in data recorded by the extramedullary cuff electrode. The frequency, timing, and amplitude of the identified artifacts exactly mirrored the preprogrammed stimulation parameters delivered at the time of recording. Furthermore, an increase in signal strength was visible between the extramedullary recording electrode and the intramedullary recording electrode, communicating that the artifact is not the result of a malfunctioning implant, but the product of a discrete electrical field generated on demand at the location of our stimulating cuff electrode (Figure 6). This collection of evidence validates our system’s ability to remotely stimulate and record from our sheep subject’s nervous system four weeks after implantation. No evidence of a stimulation-evoked compound action potential was recorded and no behavioral response to stimulation was observed.

**FIGURE 7:**
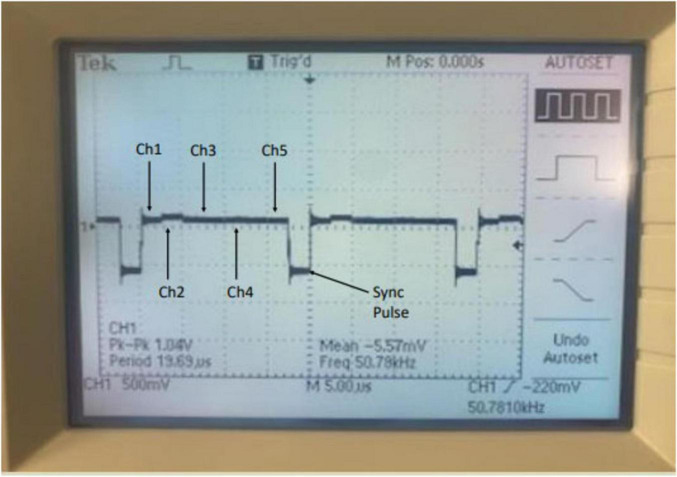
Oscilloscope reading of the receiver sync pulse taken at 8 weeks demonstrating the recording capsule is functional and successfully communicating with the receiver box. Time division multiplexing enables transmission of data from all 5 Channels at 50 k Hz sampling per channel of the recording implant through one multiplexed output signal. Information from each channel is split sequentially between the sync pulse.

Generation of a synchronization pulse via oscilloscope enabled validation of our remote recording-receiver system. Figure 7 illustrates a properly functioning recording implant; the synchronization pulse imaged in the figure serves as the anchor point for the near simultaneous transmission of five concurrent data streams captured by the recording electrode. Generated 8 weeks post-implantation, a proper synchronization pulse from our recording implant communicates sustained viability of our capsule design and telemetric approach throughout the experimental period.

**FIGURE 8:**
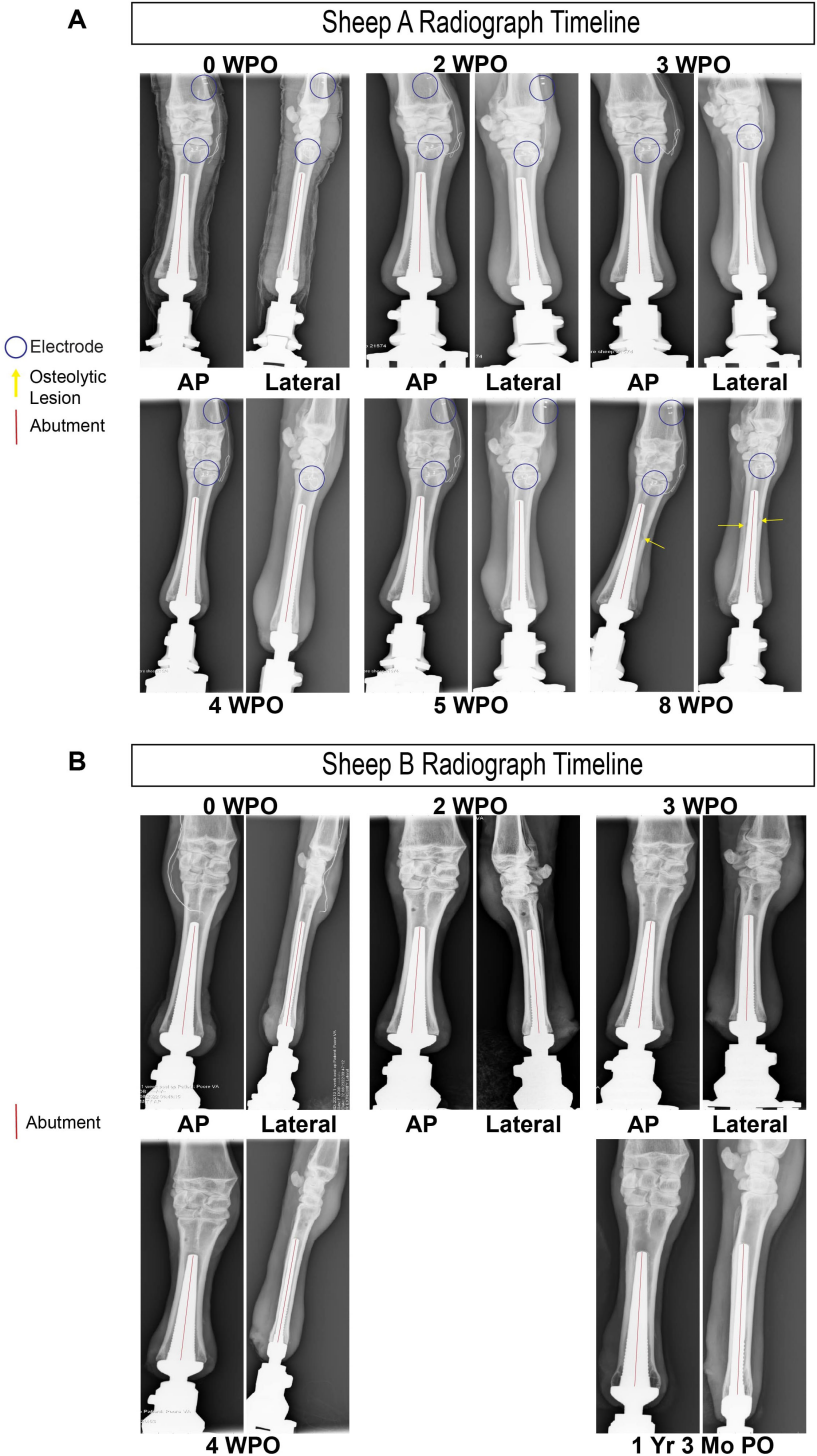
**(A)** Radiological imaging of Sheep A from 0 weeks post-op (WPO) to 8 WPO **(B)** radiological imaging of Sheep B from 0 WPO to 1 year and 3 months post-op.

At approximately 9 weeks post-operation, lameness appeared in Sheep A. Following immediate consultation with the veterinary orthopedic surgeon, the primary diagnosis of osteomyelitis was made via radiological imaging (Figure 8A, yellow arrow), and the decision was made to immediately euthanize based on recommendation. A full necropsy was performed by a board-certified veterinary pathologist and soft tissue, bone, and implant material samples were all taken from several areas and both aerobic and non-aerobic cultures came back negative. Complete blood count (CBC) was unremarkable. Histology showed increased bone remodeling. The bone lysis seen on radiographs was not due to an infection, but possible osteonecrosis or avascular ischemia. Further studies are needed to determine the cause.

## Discussion

We demonstrate the feasibility of a wireless Ovine Osseointegrated Neural Interface (ONI) model, supported by radiological evidence from Sheep A and Sheep B (Figure 8B), as well as electrophysiological evidence collected exclusively from Sheep A. The model is the product of osseointegration, a refined surgical methodology, and a dual capsule implantable neural interface capable of wirelessly transmitting neural recording data to an external DAQ system. The wireless interface functioned reliably over the study period, illustrated by the presence of synchronization pulses 8 weeks post-surgery. Despite this, an idiopathic and progressive osteolytic lesion required premature euthanasia prior to telemetric failure to prevent untreatable pain and discomfort. Future goals of this model involve additional design optimizations such as advancing to a closed loop system to enhance interface functionality and expanding to a truly bidirectional design.

Neural control of a sophisticated prosthesis requires a robust connection with the patient’s nervous system, enabling seamless control of their artificial limb. As the push for greater degrees of interface grows, engineers and prosthetists must confront the decision to sacrifice stability for selectivity; surface EMGs are exchanged for percutaneous leads and the risk for tissue damage or adverse immune reactions increases (Ghafoor et al., 2017). This reciprocal exchange of stability for selectivity has produced an impasse in the development of neural prosthetics, requiring researchers to devise innovative methods in order to access relevant neural tissue without compromising the integrity of surrounding biology. Further complicating this conundrum, improper management of residual nerves will prompt the formation of painful neuromas (Adewole et al., 2016). Despite regenerating nervous tissue providing an ideal target for maximizing interface selectivity, the settled upon method of interface must involve an effective reemployment of transected nerves through provision of sufficient end targets for reinnervation (Hwang et al., 2024). Finally, high density neural recording data must be effectively exported to a computation platform capable of decoding user intention into prosthetic action. Wired systems, while capable of high-fidelity transmission, obstruct natural movements of the organism, impeding study of how neural interfaces impact the behavior of a freely ambulating animal. By extension, the ideal peripheral nerve interface should balance the need for selectivity and stability, circumvent neuroma formation, and transmit dense recording data without impairing the organism’s mobility.

An Osseointegrated Neural Interface is one approach capable of meeting these requirements, enabling the isolation of target nerves within an environment that supports their function and minimizes the interference of implanted electronics with more reactive soft tissue (Dingle et al., 2020). Housing a neural interface within long bones also opens the door to interfacing methods previously considered too invasive for a percutaneous approach; sieve electrodes, for example, enable the isolation of individual fascicles for recording or stimulation, but have often been precluded from interface design due to their fragile nature and prerequisite of neurotmesis (Coker et al., 2019). Incorporation into an Osseointegrated Neural Interface, however, would shield these sensitive devices from external disturbances, achieving both selectivity and stability (Sears et al., 2024). Furthermore, implantation of a transected nerve into the medullary canal of a long bone is a clinically viable approach to managing symptomatic neuromas (Israel et al., 2018). The ONI is designed around these advantages of osseointegration with the end goal of deploying a longitudinally stable peripheral nerve interface capable of closed-loop bidirectional control of an endoprosthetic abutment.

Currently, our Ovine ONI is an experimental alternative to this ideal; the dual implantable capsule design enables real-time, remote stimulation and recording from our subject’s nervous system. Evidenced by the presence of artifact originating from our stimulation electrode that mirrored preprogrammed parameters in our recording data, this system is capable of remotely delivering a wide range of stimulation patterns to implanted electrodes. These patterns and any bioelectric responses can then be detected by the five recording electrodes, whose signals are multiplexed and simultaneously transmit to receivers external to the animal. The wireless nature of this system allows for close behavioral observation of the animal subject; unimpeded by bulky electronics or obtrusive wires, adverse or positive reactions can be more directly attributed to activity of the neural interface. Furthermore, our radiological evidence supports the mechanical viability of the Ovine ONI; the endoprosthesis was stably embedded into the medullary canal without fracture and the electronics remained in their optimal locations despite residing in a freely ambulating animal. In addition to their mechanical stability, the synchronization pulse generated eight weeks post-implantation communicates that our implants are functionally stable for a prolonged experimental period, as any infiltrating moisture would quickly disable the sensitive components housed within the capsules. Taken in concert, this evidence demonstrates a stable peripheral nerve interface capable of wireless stimulation and recording from a freely ambulating animal subject’s nervous system.

Critical next steps of the Ovine ONI would involve incorporating a closed-loop approach to peripheral nerve interface design, in which a force-sensitive prosthetic would communicate with the stimulation capsule, engaging the animal’s somatosensory system in a more organic fashion. Coupling this closed-loop approach with a more selective interface incorporating sieve electrodes would enable the construction of an Ovine ONI that can encode afferent sensory input and decode efferent sensory output for a truly bidirectional peripheral nerve interface. Considering our wireless system’s longitudinal viability and intrinsic value for behavioral study, one could characterize the long-term impact of a bidirectional, neurally integrated prosthetic device in a large animal model. Sheep B demonstrates the surgical approach and chronic capacity of this model for future open- and closed-loop experiments. The changes in bone morphology seen in Sheep A necessitates further studies to determine the cause.

## Conclusion

Here, we demonstrate the viability of our Ovine ONI model complete with a wireless, dual capsule implantable peripheral nerve interface. Our electrophysiological and radiological evidence emphasizes the potential of osseointegration to innovate the field of sophisticated prosthesis design and communicates that nerves transposed into a medullary canal can serve as viable targets for stimulation and recording. Furthermore, this study will serve as the foundation for future iterations of the ONI, aiming to achieve a closed-loop bidirectional system for close study of the longitudinal implications of a neurally active prosthesis. Overall, these findings provide an early methodological foundation that may help address longstanding challenges in neural interfacing by providing a stable platform for neural recording and stimulation. By leveraging osseointegration to enhance prosthetic control and sensory feedback, this model lays the groundwork for future advancements in peripheral nerve interface design, ultimately bringing neural prostheses closer to seamless integration with the human nervous system.

## Data Availability

The datasets presented in this study can be found in online repositories. The names of the repository/repositories and accession number(s) can be found below: https://github.com/lucassears2000-ui/ONI_Frontiers_Submission_10_12.git.
